# Association of serum total bilirubin levels with diastolic dysfunction in heart failure with preserved ejection fraction

**DOI:** 10.1186/0717-6287-47-7

**Published:** 2014-03-26

**Authors:** Huan Zheng, Ye Li, Nanzi Xie

**Affiliations:** Geriatrics Department, Tongji Hospital Affiliated to Tongji University, 389 Xincun Road, Shanghai, 200065 P.R. China; Heart Failure Department, Shanghai East Hospital Affiliated to Tongji university, Shanghai, China

**Keywords:** Bilirubin, Heart failure, Diastolic dysfunction, Echocardiography

## Abstract

**Background:**

Left ventricular diastolic dysfunction is one of the main characteristics of heart failure patients with a preserved left ventricular ejection fraction. As bilirubin is regarded as an important endogenous antioxidant molecule, serum total bilirubin levels were compared between heart failure patients with a preserved left ventricular ejection fraction and normal controls in this study. We recruited 327 heart failure patients with a preserved left ventricular ejection fraction and 200 healthy controls. Patients were divided into 4 subgroups by their comprehensive echocardiographic manifestations, 1-mild, 2-moderate, 3-severe (reversible restrictive), 4-severe (fixed restrictive). Total bilirubin levels were compared using stepwise multiple regressions adjusted for selected factors.

**Results:**

After adjusting for gender, age, smoking, systolic blood pressure, diastolic blood pressure, total cholesterol and triglyceride, serum total bilirubin levels were significantly lower in the heart failure group compared with the control group (P < 0.01). Patients in the subgroup (4-severe) showed significantly (P < 0.05) lower levels of total bilirubin when compared with the subgroup (1-mild).

**Conclusions:**

TB level was negatively correlated with left ventricular diastolic dysfunction in heart failure patients with a preserved left ventricular ejection fraction, which might provide a new insight into the complicated mechanisms of heart failure with a preserved left ventricular ejection fraction.

## Background

Bilirubin, once considered simply as the metabolic end-product of heme degradation, has now emerged as an important endogenous antioxidant molecule. Earlier researches have demonstrated an inverse correlation between serum bilirubin and the risk of coronary artery disease and decreased antioxidant activity of bilirubin in atherosclerotic lesions [1, 2]. Recent studies have confirmed the correlation between serum bilirubin levels and collateral development in patients with chronic total coronary occlusion [3], peripheral arterial disease [4], amputation events in type-2 diabetes mellitus [5], stroke severity and clinical outcomes [6]. Those results suggested that bilirubin may be a part of a cell defense strategy in those macro- and micro- angiopathies.

Heart failure with a preserved left ventricular ejection fraction (HFpEF) is a clinical syndrome characterized by the symptoms and signs of heart failure (HF), a preserved left ventricular ejection fraction (LVEF ≥ 50%) and abnormal diastolic function [7]. The percentage of patients with HFpEF in epidemiological studies ranges from 40% to 71% (mean 56%), but in hospital-based cohort studies it is slightly lower, ranging from 24% to 55% (mean 41%) [8]. Despite the better LVEF, HFpEF has a prognosis similar to that observed in HF patients with reduced LVEF, with a mortality rate exceeding 20% in 1 year [9]. More and more studies have suggested that chronic HF may be potentially characterized as an oxidative disease [10–12]. Recently, Radovanovic et al. showed that by-products of oxidative damage and antioxidant enzyme activities were correlated with echocardiographic indices of remodeling. After a long-term follow-up, they found that the level of malondialdehyde, which was a biomarker of lipid peroxidation, might be a useful parameter for monitoring and planning management of chronic HF patients [13].

Diagnostically, echocardiography is an important tool used to identify HFpEF and to stratify impaired diastolic function according to its level of severity (mild, moderate and severe) [14]. Multiple studies revealed a correlation between the severity of diastolic dysfunction and risk of death [15, 16]. Unlike the association between serum total bilirubin (TB) levels and vascular events, which has been widely studied, whether or not TB plays a role in HFpEF is not yet clear. This study was designed to determine whether TB levels were significantly different in HFpEF patients compared with controls and to investigate the association between TB levels and echocardiographic diastolic dysfunction grades.

## Results

Characteristics of all the HFpEF patients and healthy controls were shown in Table 1. The 327 HFpEF patients were categorized into four subgroups according to their comprehensive echocardiographic manifestations. The HFpEF group included 130 males and 197 females with a mean age of 66.8 ± 10.3 years, and the control group consisted of 116 males and 84 females with a mean age of 43.6 ± 9.7 years. In the HFpEF group, 39.8% of the participants were male and in the control group 58% were male (*P* < 0.01). Age, body mass index, systolic blood pressure, diastolic blood pressure, total cholesterol, low-density lipoprotein, triglyceride and creatinine levels were all significantly greater in the HFpEF group than in the control group (*P* < 0.05). There were no differences between the two groups with respect to high-density lipoprotein level, fasting glucose, alanine aminotransferase and urea level (*P* > 0.05).

**Table 1:** **Characteristics of the controls and HFpEF patients**

	Control group (n = 200)	HFpEF groups				
		1-mild (n = 52)	2-moderate (n = 116)	3-severe (reversible restrictive) (n = 138)	4-severe (fixed restrictive) (n = 21)	***P*** ^*^-value
Male (n, %)	116 (58%)	31 (5.6%)^a^	44 (37.9%)^a^	49 (35.5%)^a^	6 (28.6%)^a,b,c,d^	0.003
Age (years)	43.6 (9.7)	69.7 (7.3)^a^	65.9 (10.2)^a^	67.1 (11.4)^a^	66.3 (13.1)^a^	0.001
BMI (kg/m^2^)	22.7 (2.5)	24.3 (3.6)^a^	23.9 (4.7)	24.5 (2.1)^a^	23.8 (3.7)	0.048
Smoker (n, %)	85 (42.5%)	25 (48.1%)	39 (33.6%)	38 (27.5%)^a^	5 (23.8%)^a,b,c^	0.028
Medications						
ASA (n, %)	0	48 (92.3%)^a^	110 (94.8%)^a^	134 (97.1%)^a^	20 (95.2%)^a^	<0.001
Diuretic (n, %)	0	21 (40.4)^a^	64 (55.2%)^a,b^	70 (50.7%)^a,b^	18 (85.7%)^a,bc,d^	<0.001
Beta blocker (n, %)	0	21 (40.4%)^a^	44 (37.9%)^a^	50 (36.2%)^a^	8 (38.1%)^a^	<0.001
ACEI/ARB (n, %)	0	47 (90.4%)^a^	112 (96.6%)^a^	133 (96.4%)^a^	21 (100%)^a^	<0.001
Statin (n, %)	0	50 (96.2%)^a^	111 (95.7%)^a^	132 (95.7%)^a^	19 (90.5%)^a^	<0.001
CCB (n, %)	0	27 (51.9%)^a^	52 (44.8%)^a^	66 (47.8%)^a^	9 (42.9%)^a^	<0.001
HR (b/min)	77.4 (6.1)	75.8 (6.7)	78.2 (7.1)	71.1 (5.8)^a^	80.2 (7.2)^d^	0.036
SBP (mmHg)	120.1 (8.6)	124.6 (5.8)	138.1 (10.1)^a,b^	140.2 (9.3)^a,b^	150.6 (7.2)^a,b,c^	0.005
DBP (mmHg)	78.2 (7.0)	80.3 (8.8)	81.2 (6.5)	90.2 (10.3)^a,b,c^	91.4 (9.5)^a,b,c^	0.017
Total cholesterol (mg/dl)	191.7 (19.1)	209.3 (20.2)^a^	234.7 (19.0)^a^	225.6 (18.8)^a^	242.5 (22.7)^a^	0.007
LDL (mg/dl)	108.3 (17.4)	124.1 (18.2)^a^	120.6 (19.1)^a^	125.7 (18.8)^a^	131.9 (21.2)^a^	0.004
HDL (mg/dl)	51.3 (7.5)	43.4 (8.2)	44.0 (8.1)	47.9 (6.3)	45.2 (7.7)	0.069
Triglyceride (mg/dl)	128.8 (19.4)	150.9 (17.3)^a^	152.6 (18.4)^a^	147.6 (20.5)^a^	144.1 (19.6)^a^	0.025
Fasting glucose (mg/dl)	95.2 (7.3)	96.3 (8.1)	100.7 (6.8)	94.9 (7.4)	102.5 (8.2)	0.326
Creatinine (umol/L)	83.6 (10.7)	92.4 (11.1)	95.3 (9.6)	121.3 (12.5)^a,b,c^	127.6 (13.5)^a,b,c^	0.013
Urea level (umol/L)	261.4 (23.9)	252.4 (22.1)	274.5 (19.3)	282.6 (20.7)	288.2 (21.6)	0.204
Alanine aminotransferase (U/L)	25.4 (6.4)	32.1 (7.9)	29.6 (8.1)	30.1 (6.4)	32.1 (7.5)	0.338
TB (umol/L)	15.3 (4.1)	14.1 (3.2)	12.5 (3.6)^a^	11.8 (2.9)^a^	10.4 (4.0)^a,b^	0.001

Values are mean ± SD or numbers. BMI, body mass index; HR, heart rate; ASA: acetylsalicylic acid; ACEI: angiotensin-converting enzyme inhibitor; ARB: angiotensin II receptor blocker; CCB: calcium channel blocker; SBP, systolic blood pressure; DBP, diastolic blood pressure; LDL, low density lipoprotein; HDL, high density lipoprotein; TB, total bilirubin; Numerical variables were compared by *t*-test; Categorical variables (male/female, smoker, medications) were compared by chi-square test. *P* ^*^-value, control group vs. HFpEF group; ^a^ *P* < 0.05 compared with control, after Bonferroni correction; ^b^ *P* < 0.05 compared with stage 1-mild, after Bonferroni correction; ^c^ *P* <0.05 compared with stage 2-modeate, after Bonferroni correction; ^d^ *P* < 0.05 compared with stage 3-severe (reversible restrictive), after Bonferroni correction.

Since significant differences were found between the HFpEF group and the control group for the association of some variables and serum TB levels, differences in TB level between the HFpEF and control group were examined after adjusting for gender, age, smoking, systolic blood pressure, diastolic pressure, total cholesterol and triglyceride. A significantly lower serum level of TB was found in the HFpEF group compared with the control group after adjustment (*P* < 0.01). In addition, the level of TB was significantly lower in patients with grade 4 than those with grade 1 disease (*P* < 0.05).

Simple linear regression and multivariate regression with stepwise selection were used to evaluate factors associated with TB level (Table 2). During the stepwise selection process, we adopted the approach of bidirectional elimination, testing at each step for variables to be included or excluded (α_in_ = 0.05, α_out_ = 0.10). Results showed that gender, age and diastolic dysfunction echocardiographic grading were significantly associated with TB level (*R*^*2*^ *=* 0. 261, *P* < 0.05).

**Table 2:** **Results of univariate and multivariate regression with stepwise selection estimating TB levels**

Varibles	Univariate			Multivariate		
	β	SE(β)	***P***	β	SE(β)	***P***
Male	1.62	0.32	0.002	3.47	0.83	0.001
Age	0.06	0.02	0.021	0.09	0.04	0.003
BMI	0.24	0.06	0.375			
Smoke	-0.57	0.39	0.427			
Total cholesterol	-0.38	0.14	0.224			
HDL cholesterol	0.69	0.41	0.573			
LDL cholesterol	-0.51	0.10	0.106			
Triglyceride	0.82	0.37	0.483			
SBP	-0.12	0.06	0.504			
DBP	-0.08	0.14	0.669			
Diastolic dysfunction grade	-0.92	0.35	0.037	-1.03	0.39	0.021

BMI, body mass index; HDL, high density lipoprotein; LDL, low density lipoprotein; SBP, systolic blood pressure; DBP, diastolic blood pressure; β, standardized regression coefficient; SE(β), standard error of the standardized regression coefficient. Correlation between the continuous variable and TB level was made by Pearson’s method; correlation between the discrete variable and TB level was made by Spearman’s method; Multivariate regression model: α_in_ = 0.05, α_out_ = 0.10, R^2^ = 0.261.

## Discussion

In this study, none of the HFpEF patients had any relevant diseases that could reduce the serum TB level, and we found that serum TB levels were significantly lower in HFpEF patients than those in normal controls after adjusting for age, gender, body mass index, smoking, systolic blood pressure, diastolic blood pressure, total cholesterol and triglyceride. In addition, the serum TB levels in HFpEF grade 4 (severe, fixed restrictive) was significantly lower than those in HFpEF grade 1 (mild). Last but not least, HFpEF echocardiophic grading was significantly associated with serum TB levels in simple linear regression and multivariate regression analysis.

### Pathophysiology of HFpEF

Recent epidemiological studies have provided growing recognition that HFpEF is a common and costly clinical entity that is increasing in prevalence [8, 9]. The pathophysiology of HFpEF is distinct from that of HF with reduced LVEF and is characterized by left ventricular (LV) diastolic dysfunction [17]. Echocardiography is an essential non-invasive tool useful for diagnosing and classifying LV diastolic dysfunction. A key component of LV diastolic dysfunction is LV stiffness causing inability to dilate sufficiently to cope with the venous supply from the lungs. This stiffness can be increased by any pathological process affecting the heart muscle, the most common causes being LV hypertrophy and/or myocardial ischemic lesions [18]. In addition, stiffness is aggravated by changes in extracellular matrix collagen deposition, and this can even precede LV hypertrophy [19]. Meanwhile, it has been pointed out that free radicals can be involved in cardiomyocyte hypertrophy, apoptosis and mechanisms of cardiac remodeling [10, 11]. Giordano supported the concept of reactive oxygen species contributing to the cardiac remodeling processes in a number of ways, including activating matrix metalloproteinases that participate in reconfiguration of the extracellular matrix, acting as signaling molecules in the development of compensatory hypertrophy and myocyte loss via apoptosis or other cell death mechanisms. The role of oxidative stress in LV diastolic dysfunction is very complex and those concrete pathways are still unclear; however, existence of systemic oxidative stress in chronic HF and its gradual increase as chronic HF progresses have been confirmed [13]. In recent years, the improved treatment strategy has increased survival rate in systolic HF, whereas the prognosis for HFpEF remains unchanged [9]. Risk stratification is an integral part of management in patients with HFpEF, and is also an important step in defining the optimal treatment strategy. This is why researches are searching for new biomarkers to help probe the complicated pathophysiology of HFpEF.

### Antioxidant effects of TB

Since the report in 1994 by Schwertner et al. which indicated that serum bilirubin level was negatively correlated with coronary artery disease, many studies have observed that in the general population, upper normal or modestly elevated bilirubin levels are associated with improved outcomes, such as a lower risk of coronary heart disease events, respiratory disease, and all-cause mortality [20–22]. A previous study showed that serum bilirubin levels were inversely correlated with the superoxide anion radicals generated by mononuclear cells in cardiac syndrome X patients [23]. In 2012, a study on a large statin-treated cohort reported that serum bilirubin level measured before a statin prescription to assess liver function was an independent risk factor for cardiovascular disease and death in both men and women [21]. It showed that the incidence of cardiovascular disease in the lowest decile category of bilirubin was 215 per 10,000 person-years compared with 163 per 10,000 person-years in the highest decile. As for the relationship between bilirubin and HF, the conclusions are still controversial. For example, a recent analysis of the Efficacy of Vasopressin Antagonism in Heart Failure Outcome Study with Tolvaptan (EVEREST) cohort, which included patients hospitalized with systolic HF, showed that an elevated baseline TB level and in-hospital increase in bilirubin were associated with an increased risk of all-cause mortality and combined cardiovascular mortality [24]. While in another study which focused on the relationship between liver function abnormalities and hemodynamic profile, it turned out that the predictors of all-cause mortality were γ-glutamyl transpeptidase, alkaline phosphatase, aspartate aminotransferase and lactic dehydrogenase, other than TB [25]. However, there were fewer data investigating the relationship between TB and diastolic dysfunction. In our study, serum TB level in HFpEF was lower than that in the normal controls, and serum TB level in grade subgroup 4 was lower than that in grade subgroup 1. To our knowledge, this is the first study to investigate the association between serum TB level and diastolic dysfunction grading in HFpEF. We postulated those results might be related to the antioxidant effects of bilirubin. As we all know, HF may cause hepatic congestion and reduced forward flow, which would lead to elevated TB levels, but we found that it was negatively correlated with the severity of our patients’ LV diastolic dysfunction. This finding implied that some potential benefits might outweigh the underlying severity of HF causing the TB level increasing. The mechanisms linking low bilirubin and risk or worse outcomes for cardiovascular disease are not fully understood. A number of mechanisms has been proposed for the antioxidant effects of bilirubin, including suppression of low-density lipoprotein oxidation [26], monocyte migration [27], vascular smooth muscle cell proliferation [28] and endothelial dysfunction [29]. Early animal experiments indicated that bilirubin served as a physiological antioxidant in ischemia–reperfusion [30] and served a protective function against injury-mediated proliferation of intimal cells [31]. It was reported that moderate hyperbilirubinemia reduced blood pressure in angiotensin II-dependent hypertension through decreasing oxidative stress and increasing nitric oxide levels [32]. Our study was only a clinical observation, further experiments are needed to explore the underlying mechanisms.

### Biomarkers of HFpEF

The best characterized biomarkers in patients with HFpEF are the natriuretic peptides: B-type natriuretic peptide (BNP) and N-terminal pro-BNP (NT pro-BNP) [29]. BNP is mainly released in response to increased cardiac volume and pressure overload. It is synthesized as an inactive pro-hormone that is split into the active hormone BNP and the inactive NT pro-BNP. Both elevated NT pro-BNP and BNP are strong independent predictors of clinical events in patients with HFpEF. No consensus exists on use of natriuretic peptide levels to guide medical therapy [33]. Our study was an attempt to invesigate whether bilirubin might play an role in the pathogenic processes that link oxidative stress and diastolic dysfunction in HFpEF patients.

### Limitations

There were potential limitations to this study. First, the sample size was relatively small. Further studies with larger sample sizes are needed to confirm our results. Second, our study was cross-sectional, and it would have been ideal to have obtained repeated determinations in the same patients over time in our study to prove their relationship.

## Conclusions

The results of this study indicated a correlation between decreased serum TB level and increasing severity of LV diastolic dysfunction in HFpEF. Further in vivo studies investigating the mechanisms underlying decreased TB in patients with HFpEF may assist in developing new targets for prevention and treatment.

## Methods

### Subjects

This observational study included 327 patients (130 male and 197 female, aging 66.8 ± 10.3 years old) with HFpEF who were hospitalized in our department during the period from January 2008 to January 2013 and a normal control group consisting of 200 healthy volunteers (116 male and 84 female, aging 43.6 ± 9.7 years old) who attended an annual checkup at our hospital in 2012. HFpEF was diagnosed according to criteria in the consensus statement of the Heart Failure and Echocardiography Associations of the European Society of Cardiology [7]. Patients with systolic HF, severe valvular stenosis or regurgitation, congenital heart disease, atrial fibrillation, chronic obstructive pulmonary disease, malignancy, other extracellular fluid-increasing diseases such as hypothyroidism, diabetes, serious cerebrovascular diseases, a history of liver or gallbladder or bile-duct disease, hemolytic disease, rheumatic or immune diseases were excluded. The control participants had no cardiovascular or any other organ system disease, and had normal physical examination, chest roentgenogram, electrocardiogram, two-dimensional and Doppler echocardiogram. All participants gave their informed consent, and the study protocol was approved by ethical review board of Tongji Hospital Affilated to Tongji University, Shanghai, China.

### Laboratory measurement

5 ml venous blood sample was collected from each participant under fasting conditions. Then they were centrifuged and the serum was extracted for use, with spinning speed 800 g for 5 mins at 4°C and storage temperature -80°C. Fasting blood glucose, total cholesterol, high-density lipoprotein cholesterol, low-density lipoprotein cholesterol, triglyceride, uric acid, creatinine and TB levels were recorded. TB levels were analyzed using an autoanalyzer (Roche Diagnostic Modular Systems, Tokyo, Japan).

### Echocardiography

All participants underwent complete transthoracic echocardiographic studies (Vivid 7 system, General Motor Corp., USA) using a 2.5–4.0-MHz transducer. LVEF was measured using Simpson’s method. The following conventional mitral inflow pulse wave Doppler parameters were measured: peak early (E) and late diastolic (A) transmitral filling flow velocities, E/A ratio, ∆E/A with a Valsalva maneuver and deceleration time (DT) of the E wave and isovolumic relaxation time (IRT), defined as the time from aortic valve closure to mitral valve opening. Pulmonary venous flow parameters were also measured: peak systolic velocity (Ps), peak diastolic velocity (Pd), Ps/Pd ratio, atrial reversal (PVAr) velocity, the difference in PVAr and transmitral A velocity duration (PVAr-A). Tissue Doppler parameters were measured: peak systolic mitral annular velocity (S’), early diastolic mitral annular velocity (E’) and late diastolic mitral velocity (A’). Those parameters were obtained from the apical four-chamber view, 1–3-mm sample volume for mitral leaflets, 2–3-mm for pulmonary vein, 2–5-mm for mitral leaflets, respectively. The mean of three measurements was used for analysis of the Doppler data. The ratio of mitral peak velocity of early diastolic filling to early diastolic mitral annular velocity (E/E’) was calculated for the lateral and septal annulus. All the HFPEF patients were divided into four groups: 1 – mild, 2 – moderate, 3 – severe (reversible restrictive), 4 – severe (fixed restrictive) [14]. 1 (mild): E/A <0.8, ∆E/A (Valsalva maneuver) <0.5, DT > 200 ms, E/E’lateral < 8, Ps > Pd, PVAr-A < 30 ms; 2 (moderate): 0.8 < E/A < 2, ∆E/A (Valsalva maneuver) ≥ 0.5, 160 < DT < 200 ms, E/E’lateral ≥ 8–12, Ps < Pd, PVAr-A ≥ 30 ms; 3 (severe) (reversible restrictive): E/A ≥ 2, ∆E/A (Valsalva maneuver) ≥ 0.5, DT < 160 ms, E/ E’lateral > 12, Ps < Pd, PVAr-A ≥ 30 ms; 4 (severe) (fixed restrictive): E/A ≥ 2, ∆E/A (Valsalva maneuver) < 0.5, DT < 160 ms, E/ E’lateral > 12, Ps < Pd, PVAr-A ≥ 30 ms.

### Statistical analysis

All statistical analyses were carried out using statistical software (SPSS, version 15.0 for Windows; SPSS Inc., USA). All numeric variables were expressed as mean ± SD and normally distributed. We performed a hierarchal analysis between two groups (controls and HFpEF) and then a further analysis among 5 groups (Control, 1-mild, 2-moderate, 3-severe reversible, 4-severe fixed) with Bonferroni-correction. Numerical variables were compared by *t*-test and categorical variables were compared by chi-square test. Simple linear regression and multivariate regression with stepwise selection were used to evaluate factors associated with TB levels. Probability values of *P* < 0.05 were considered significant.
